# A Case of Piperacillin-Induced Immune Thrombocytopenia: Diagnostic Challenges and Management

**DOI:** 10.7759/cureus.94174

**Published:** 2025-10-09

**Authors:** Arpeet Patel, Kuldeepsinh Atodaria, Andrew C Rettew, Niharika Kottapalli, Pratap Tetali

**Affiliations:** 1 Radiation Oncology, Drexel University College of Medicine, Philadelphia, USA; 2 Hematology and Oncology, Tower Health Medical Group, West Reading, USA

**Keywords:** drug hypersensitivity, drug-induced adverse reaction, drug-induced immune thrombocytopenia, piperacillin, thrombocytopenia

## Abstract

Drug-induced immune thrombocytopenia (DITP) is a rare yet critical disorder that requires prompt recognition and discontinuation of the causative drug to prevent severe complications. In DITP, platelet-reactive antibodies lead to significant platelet destruction. Heparin-induced thrombocytopenia is the most well-studied; yet, antibiotics have also been described.

Herein is a case of a 97-year-old woman with a history of hypertension and hyperlipidemia who was admitted with rhabdomyolysis and sepsis secondary to choledocholithiasis and gallstone pancreatitis. She was started on intravenous fluids and broad-spectrum antibiotics, including piperacillin-tazobactam and vancomycin. Within days, her platelet count dropped from 323,000/μL to 1,000/μL. Schistocytes and hemolysis were absent on a peripheral smear. The patient had normal coagulation studies, and she had a low 4T (thrombocytopenia, timing of platelet count fall, thrombosis or other sequelae, and other causes for thrombocytopenia) score, ruling out thrombotic microangiopathies, such as disseminated intravascular coagulation (DIC), thrombotic thrombocytopenic purpura (TTP), and heparin-induced thrombocytopenia (HIT), respectively. While immune thrombocytopenic purpura (ITP) was seriously considered, the temporal relationship between the drop in platelet count and the administration of broad-spectrum antibiotics led to greater suspicion of DITP. Piperacillin was suspected to be the cause and was promptly discontinued. The suspicion was subsequently confirmed, as supported by the detection of positive drug-dependent IgG antibodies. The patient’s platelet count normalized within a week after stopping piperacillin and receiving IV immunoglobulin (1 g/kg). Hence, DITP needs to be considered among other causes in patients with acute severe thrombocytopenia, as early recognition and prompt cessation of the offending agent are important for preventing life-threatening hemorrhagic sequelae.

## Introduction

Drug-induced immune thrombocytopenia (DITP) is a rare, yet serious, condition that requires prompt recognition and withdrawal of the offending medication to prevent significant bleeding complications and mortality from occurring. DITP usually occurs within 5-10 days after exposure to the inciting medication, but it can develop within hours if there has been prior sensitization [1]. The rapid fall in platelet count with DITP occurs due to platelet destruction, with the formation of antibodies. While the mechanism is not fully understood, a minimum of three types of antibodies factor into the severe platelet destruction: hapten-dependent antibodies, drug-induced platelet-reactive antibodies, and drug-dependent antibodies. Beta-lactam antibiotics, such as cephalosporins and penicillins, are rare, inciting drugs that can form allergens primarily by forming hapten-protein complexes [2]. Medications such as procainamide and gold induce auto-antibodies that do not require the drug itself to bind to targets on the platelet membrane [3]. Most commonly, drug-dependent antibodies lead to platelet destruction by targeting glycoproteins on the membrane. However, once the drug binds to the glycoproteins, it is poorly understood how antibody binding is promoted. The most likely mechanisms are that the antibodies recognize either the “compound epitope” formed by the noncovalent bond between the drug and glycoprotein complex, or the antibodies recognize a conformational change in the glycoprotein complex incited by the drug binding to a distinct site [4].

Within the general population, the incidence is estimated to be about 10 cases per 1,000,000 per year. However, this rate is higher in those who are in the hospital or use higher-risk medications such as nonsteroidal anti-inflammatory drugs (NSAIDs) or sulfonamides [4]. Antibiotics such as ceftriaxone, vancomycin, penicillins, and macrolides have also been associated with DITP [5]. Exclusion of other causes of thrombocytopenia is crucial; the most important being idiopathic thrombocytopenic purpura (ITP), since it has been common for patients with DITP to be falsely diagnosed with ITP [6]. Other causes to rule out include disseminated intravascular coagulation (DIC), heparin-induced thrombocytopenia (HIT), thrombotic thrombocytopenic purpura (TTP), hemolytic uremic syndrome (HUS), and pseudo-thrombocytopenia, which is an in vitro effect caused by anticoagulants such as ethylenediaminetetraacetic acid (EDTA), leading to platelet clumping [4]. While laboratory testing for drug-dependent antiplatelet antibodies is not required, it helps in confirming the diagnosis [3].

DITP may lead to life-threatening, progressive reductions in platelet counts with bleeding. Generally, establishing the temporal relationship of drug exposure and thrombocytopenia is of great importance in diagnosing the cause of thrombocytopenia [7]. Arnold et al. described clinical criteria for the diagnosis of DITP, highlighting the need to rule out other causes as well as document platelet recovery after withdrawal of the offending medication [2]. We report a rare case of DITP secondary to piperacillin, confirmed with drug-dependent immunoglobulin G (IgG) antibody testing, and emphasize the importance of recognizing and treating DITP quickly to prevent life-threatening complications.

## Case presentation

**Figure 1:**
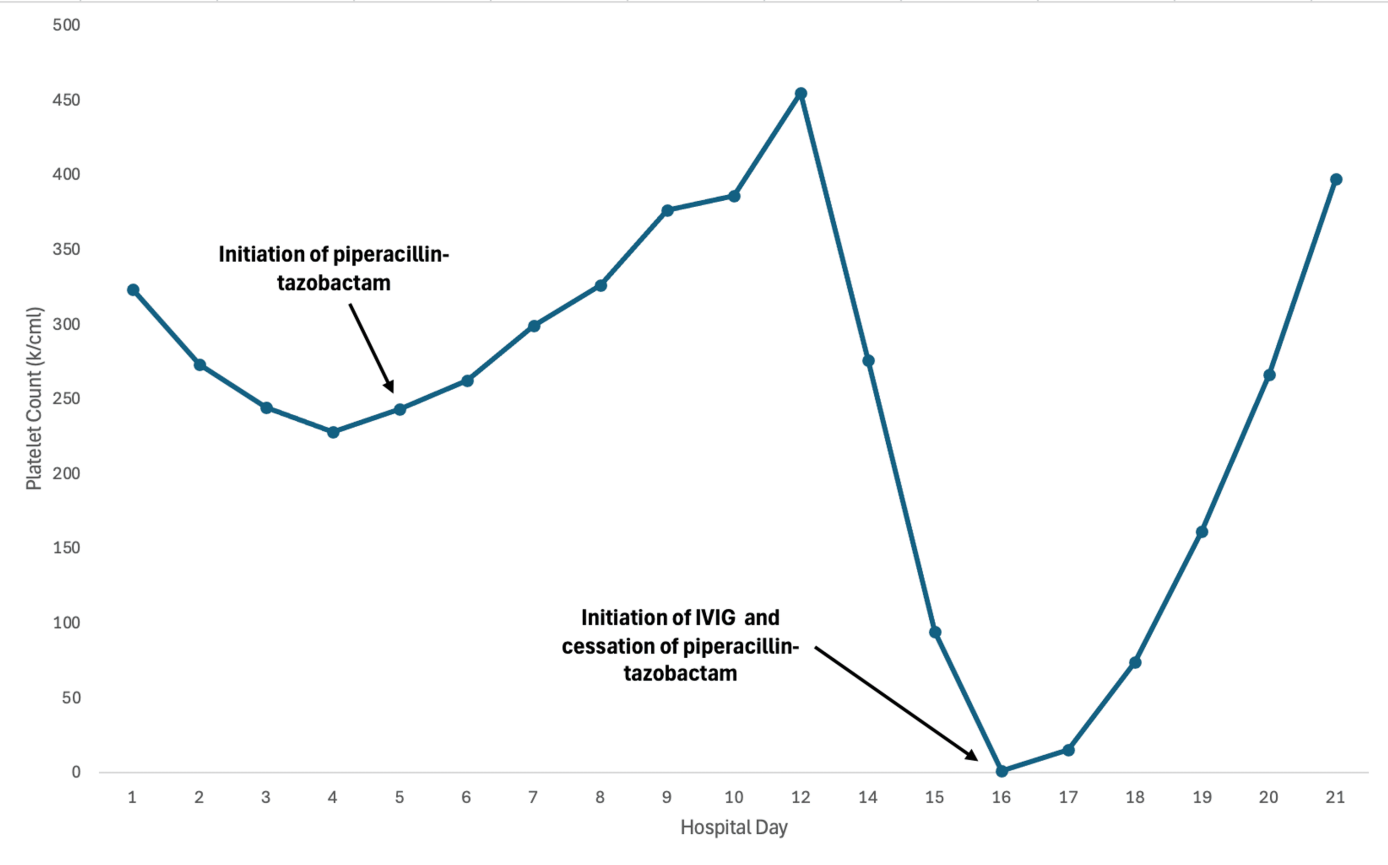
Trend of platelet count upon initiation and cessation of piperacillin-tazobactam

A 97-year-old woman with a history of hyperlipidemia and hypertension presented to the hospital after an unwitnessed fall and rhabdomyolysis, likely secondary to several days of immobility. On admission, the patient was significantly dehydrated, with hypernatremia and lactic acidosis. Subsequently, the patient was started on intravenous fluids and broad-spectrum antibiotics, including piperacillin-tazobactam and vancomycin, for a diagnosis of sepsis secondary to choledocholithiasis and gallstone pancreatitis. Her initial laboratory studies indicated a white blood cell count of 23,000/μL, hemoglobin of 15.6 g/dL, and platelets of 323,000/μL (Table 1). Over the next 11 days, her platelet count dropped to 1,000/μL (Figure 1). She had received a prophylactic dose of enoxaparin, a low molecular weight heparin, for three days upon initial admission. She was also diagnosed with acute-onset atrial fibrillation on day 4 of hospitalization, for which she was started on apixaban at a dose of 2.5 mg twice daily, adjusting the dose for her age, body weight, and renal function.

**Table 1:** Patient’s laboratory work on hospital admission day 1

Lab Value	Patient's Lab Value	Normal Range
Hemoglobin (HGB)	15.6 g/dL	14.0–17.4 g/dL
White blood cell count (WBC)	23.1 k/mcL	4.4–11.0 k/mcL
Platelet count	323 k/mcL	145–445 k/mcL
Mean corpuscular volume (MCV)	96.9 fL	80–96.0 fL
Prothrombin time (PT)	18.1 s	11.8–14.3 s
International normalized ratio (INR)	1.5 H	0.9–1.1 H
Sodium	152 mmol/L	136–145 mmol/L
Potassium	3.9 mmol/L	3.5–5.2 mmol/L
Chloride	112 mmol/L	98–107 mmol/L
CO2	25 mmol/L	22-30 mmol/L
Blood urea nitrogen (BUN)	97 mg/dL	8–23 mg/dL
Creatinine	1.14 mg/dL	0.7–1.2 mg/dL
Aspartate transferase (AST)	58 U/L	0–40 U/L
Alanine transaminase (ALT)	84 U/L	0–41 U/L

Due to the severity of the thrombocytopenia, a hematology consult was obtained. On the peripheral smear, there was no evidence of schistocytes or hemolysis. Coagulation studies resulted in normal prothrombin time (PT) and activated partial thromboplastin time (aPTT), ruling out DIC. The PLASMIC score was calculated and found to be low, making TTP rather unlikely. HIT was considered but unlikely due to no heparin exposure (although she had received prophylactic enoxaparin for three days) and a low 4T (thrombocytopenia, timing of platelet count fall, thrombosis or other sequelae, and other causes for thrombocytopenia) score. ITP and DITP were both suspected, but DITP was given more attention, given the timing and rapid progression of thrombocytopenia. A drug-dependent antiplatelet antibody test was sent, reviewing her medications, including piperacillin, cefepime, furosemide, and pantoprazole.

Meanwhile, the patient was treated with a single dose of intravenous immunoglobulin (IVIG, 1 g/kg), after which there was a dramatic increase in platelet count. IgG antibodies against piperacillin were confirmed by antibody testing, and piperacillin was added to her allergy list. Platelets recovered within one week of stopping piperacillin-tazobactam.

## Discussion

This case highlights the need to consider DITP as a diagnosis in patients with severe thrombocytopenia, specifically in the context of recent medication exposure. DITP is characterized by the rapid and significant decrease in the platelet count to below 20,000/μL, along with the presence of drug-dependent platelet-reactive antibodies [3]. The most important step is to discontinue the offending agent, which typically leads to rapid recovery of platelet count.

The underlying mechanisms of DITP vary depending on which drug is involved. A well-described mechanism is seen with quinine DITP, where DDAbs bind to platelets only in the presence of the sensitized drug, interacting directly with the complementarity-determining regions (CDRs) of the antibody. This leads to the formation of a “hybrid paratope” through structural rearrangement, allowing for binding with high specificity and affinity to glycoproteins on platelets such as GPIIb/IIIa or GB1b/IX. Another well-understood mechanism involves hapten-dependent responses, where small molecules like penicillin bind to the platelet surface proteins, resulting in the formation of a neoantigen that is recognized by specific antibodies. The recognition leads to a classic immune response that targets the drug-protein complex [8]. Piperacillin-induced thrombocytopenia is believed to occur through the hapten-dependent mechanism [3].

Diagnostic challenges are present with DITP since the diagnosis overlaps with other thrombocytopenic etiologies. Nonetheless, consideration of all etiologies is needed for a proper diagnostic workup. In the patient presented in this case, the lack of microangiopathic hemolytic anemia, normal coagulation profiles, and a rapid platelet response to IVIG support the diagnosis of DITP. Pseudo-thrombocytopenia, commonly due to EDTA-induced platelet clumping, is important to rule out through a peripheral smear or by repeating the sample in a tube containing a non-EDTA anticoagulant [4]. Our patient received a platelet smear, which ruled out this etiology. The PLASMIC score is a validated clinical tool that allows for an estimate of the likelihood that TTP is the diagnosis. In our patient, this score was low, further supporting the exclusion of TTP as the etiology of her rapid drop in platelet count. Antibody testing confirmed piperacillin in this patient as the culprit, which is important in preventing episodes of severe thrombocytopenia on re-exposure. It is important to note, however, that although drug-dependent antiplatelet antibody testing was able to confirm diagnosis, such tests are not readily available in many institutions, and hence prompt diagnosis requires clinical judgment and temporal correlation.

Drug-induced thrombocytopenia is a serious complication but nonetheless remains underappreciated in clinical practice due to its rarity. Like piperacillin, studies have found other antibiotics to lead to DITP [9]. For instance, Butt et al. reported a case of a 25-year-old who developed severe thrombocytopenia with a platelet count of 2,000/μL shortly after starting azithromycin, which resolved after withdrawal of the antibiotic [5]. In another instance, Shah et al. discussed a patient in his 60s who was given vancomycin for concerns regarding sepsis, and subsequently had thrombocytopenia with a platelet count of 11 k/mcl, which resolved with 2 doses of 1000 mg/kg IV immunoglobulin [10]. Furthermore, in cases of significant thrombocytopenia, especially with the presence of a bleeding risk, IVIG treatment may be essential. IVIG works by disrupting platelet clearance by macrophages and is typically administered at a 1-2g/kg dosage, leading to rapid improvement in platelet counts within 1-2 days in over 80% of patients [11].

It is a common practice to start corticosteroids in these patients, but there is no established benefit in patients with DITP, likely due to the underlying pathophysiology [12]. For instance, in ITP, autoantibodies directly target platelet surface antigens regardless of exposure to external triggers, leading to phagocytosis by splenic macrophages [13]. In this case, immunosuppressive medications, such as corticosteroids, are useful to reduce the production of antibodies and the clearance of platelets by macrophages. In contrast, DITP occurs due to drug-dependent antibodies that only bind to platelets in the presence of the inciting medication. Due to the specific nature of DITP, antibody binding ceases once the offending agent is identified and discontinued, allowing for the platelet count to improve rapidly without the need for immunosuppression. Furthermore, immunosuppressive medications have an inconsistent benefit in altering the course of disease, and their use must be weighed against adverse effects, such as hyperglycemia, mood disturbances, and increased susceptibility to infections [14]. Our patient was suffering from sepsis upon presentation to the hospital, and thus, corticosteroids would have limited benefit with significant adverse effects, given further susceptibility to infection. Hence, corticosteroids are reserved for circumstances when the distinction between the etiology being ITP and DITP is unclear and must be utilized carefully after weighing the adverse effects, especially in the elderly and those with concurrent infections, as in our case.

Platelet transfusions may play a role in DITP, but must be used with caution. While transfusions are commonly used when counts fall below 10-20 × 10³/μL in bone marrow suppression or thrombocytopenia related to chemotherapy administration, their role in DITP is less established. In DITP, infused platelets are often rapidly cleared by the drug-dependent antibodies that are responsible for the destruction of endogenous platelets. However, an infusion may provide a transient benefit if there is active bleeding present or if hemostatic support is needed during procedures. In addition, the transfused platelets may be beneficial in binding the circulating offending drug, accelerating drug clearance, and shortening the duration of illness [12]. 

To reiterate, DITP management consists of discontinuing the causal agent, supportive care, and potentially IVIG to improve recovery. Platelet transfusions are indicated for active bleeding, but corticosteroids are of little benefit in DITP.

## Conclusions

DITP is a potentially serious disorder requiring timely diagnosis and treatment. Elimination of the offending agent and supportive care are essential for mitigating complications and ensuring recovery. In addition, IVIG can be considered for treating patients with DITP. Antibody testing plays a significant role in confirming the diagnosis and preventing future episodes, as demonstrated in this case. In patients with acute, profound thrombocytopenia, drug-induced thrombocytopenia should be on the list of potential differential diagnoses, especially if a recent course of antibiotics such as piperacillin has been administered.
